# Psychological correlates of performance-enhancing drug use: Emotional, cognitive, and social functioning in long-term and short-term users

**DOI:** 10.3389/fpsyt.2025.1710046

**Published:** 2025-12-02

**Authors:** Metin Çınaroğlu, Eda Yılmazer

**Affiliations:** 1 Psychology Department, İstanbul Nişantaşı University, İstanbul, Türkiye; 2 Faculty of Social Science, Psychology Department, Beykoz University, İstanbul, Türkiye

**Keywords:** performance-enhancing drugs, anabolic steroids, depression, anxiety, cognitive function, social support, self-efficacy, muscle dysmorphia

## Abstract

**Introduction:**

Performance-enhancing drug (PED) use—particularly anabolic–androgenic steroids (AAS)—has expanded from competitive sport into mainstream fitness settings. Although PED use is associated with emotional, cognitive, and social difficulties, less is known about how duration of use or psychosocial factors shape these outcomes. Understanding these dynamics is essential for identifying individuals at greater risk for psychological impairment.

**Methods:**

A total of 285 adult gym-goers (87 long-term users, 95 short-term users, 103 non-users) completed validated measures of depression (BDI-II), anxiety (BAI), muscle dysmorphia (MDDI), self-efficacy (GSE), social support (MSPSS), and social functioning (SASS), along with the Stroop test assessing executive function. Group comparisons, multiple regressions, and PROCESS-based mediation and moderation analyses were conducted, controlling for demographic covariates.

**Results:**

Long-term PED users reported significantly higher depression, anxiety, and muscle dysmorphia than short-term users and non-users, and showed poorer Stroop interference performance. Mediation analysis revealed that depression and anxiety partially explained the link between PED use and poorer social functioning. Moderation results indicated that stronger self-efficacy and higher perceived social support buffered depressive and anxious symptoms among users.

**Discussion:**

Chronic PED use is associated with heightened emotional distress and reduced executive functioning, which in turn contribute to impaired social functioning. However, psychosocial resources such as self-efficacy and social support may mitigate these adverse effects. These findings underscore the need for routine psychological assessment and the development of supportive, prevention-oriented interventions for individuals engaged in PED use.

## Introduction

Performance-enhancing drug (PED) use, including anabolic–androgenic steroids (AAS), has attracted growing attention as a public health concern—not only among competitive athletes but also among recreational gym-goers (1). While global prevalence estimates vary (2, 3), AAS use appears to be more common among men (4) than women (5) and is often motivated by aesthetic goals rather than athletic performance. Recent regional evidence similarly highlights widespread anabolic steroid use among recreational gym-goers, emphasizing limited knowledge, permissive attitudes, and behavioral normalization of such practices (6). PEDs encompass a broad range of substances, including synthetic hormones, stimulants, and supplements intended to enhance muscle growth, reduce fat, or improve overall appearance (7). Among these, AAS—such as testosterone and nandrolone—are particularly prominent in bodybuilding contexts (8). Anecdotal and clinical observations suggest that many users are motivated by body image concerns rather than competitive ambition (9). Muscle dysmorphia—a subtype of body dysmorphic disorder characterized by a persistent belief of being inadequately muscular (10)—has been proposed as a psychological driver of steroid use in some individuals (11). Users reporting muscle dysmorphia symptoms often describe dissatisfaction with their appearance despite visible muscularity, and this may contribute both to the initiation and continued use of PEDs.

Research indicates that long-term PED use can be associated with a range of emotional distress (12, 13). Some users report mood-related symptoms such as depression, anxiety, and irritability, particularly during withdrawal or off cycles (14). These symptoms are thought to be linked to disruptions in hormonal regulation, though individual experiences vary. While formal diagnostic criteria for steroid dependence are not yet included in DSM-5-TR, patterns of tolerance, withdrawal, and compulsive use have been documented in a subset of users (15). Emerging evidence also points to possible cognitive effects associated with chronic steroid use (16). Some studies suggest that long-term exposure may negatively affect executive functioning (17, 18), memory (19), and attention (20), although findings remain mixed and more research is needed. Users occasionally report subjective cognitive complaints such as forgetfulness or concentration difficulties, but objective assessments of neurocognitive performance in this population are still relatively limited.

In addition to emotional and cognitive outcomes, PED use may influence interpersonal and social functioning. Reports of increased irritability, impulsivity, or social withdrawal have been noted in clinical and qualitative studies (21, 22), though these effects are not universally experienced. It has been suggested that mood disturbances may partially account for the social difficulties faced by some users, such as strained relationships or social isolation. The nature and directionality of these associations remain unclear. At the same time, not all users experience negative outcomes to the same degree. Factors such as self-esteem and perceived social support may play protective roles (23). Individuals with a strong sense of self-worth or robust support networks may be less affected by the emotional and social risks associated with PED use. Conversely, those with low self-esteem or limited social resources may be more vulnerable to emotional distress. In the present study, general self-efficacy was assessed instead of global self-esteem, as it reflects individuals’ perceived competence in managing life challenges and stressors—conceptually overlapping with but more behaviorally oriented than self-esteem—and thus serves as a more precise indicator of the protective personal resource proposed in our framework. Although self-efficacy and self-esteem are related, they are distinct: self-esteem reflects global self-worth, whereas self-efficacy indexes perceived capability to manage challenges; we therefore focus on general self-efficacy (GSE) as the measured construct. In this study, the latter construct was chosen as it offers a more behaviorally grounded index of personal resilience (24, 25).

Despite increasing attention to the psychological consequences of PED use, relatively to the best of our knowledge no studies have compared short-term and long-term users to assess the potential cumulative effects of prolonged use. Moreover, there has been limited research examining how psychosocial factors such as self-esteem and perceived support may buffer against adverse outcomes. This study seeks to address these gaps by comparing long-term users, short-term users, and non-users on measures of emotional (depression, anxiety), cognitive (executive functioning), and social (interpersonal functioning) functioning. In addition, we examine whether emotional symptoms mediate the link between PED use and social difficulties, and whether self-esteem and social support moderate the psychological impact of use.

## Methods

### Study design

This study utilized a quantitative, cross-sectional design grounded in the positivist research paradigm, aiming to investigate the emotional, cognitive, and social correlates of steroid and performance-enhancing drug (PED) use. The design allowed for comparative and predictive analyses between three distinct user groups—long-term users, short-term users, and non-users—to test a set of pre-established hypotheses. Additionally, mediator and moderator models were employed to examine the psychological mechanisms (e.g., anxiety, depression) and contextual buffers (e.g., self-esteem, perceived social support) that may influence the relationship between PED use and psychological functioning.

### Participants and recruitment

Participants in this study were adult gym-goers aged between 18 and 55, regularly attending fitness centers across various districts of Istanbul. Recruitment was conducted face-to-face by four graduate students in clinical psychology, who visited gyms located on both the European and Asian sides of İstanbul. Individuals entering or exiting the fitness facilities were approached, informed about the study’s aims, and invited to participate. Those who agreed completed a brief sociodemographic screening form on-site and provided written informed consent. Based on their self-reported PED use history and body image concerns, participants were categorized into one of three groups: long-term users (with more than three years of PED use), short-term users (with less than one year of use), or non-users (who had never used PEDs but expressed dissatisfaction with their body image). The thresholds for user-group classification (< 1 year *vs*. > 3 years) were based on both empirical and conceptual considerations. Previous studies indicate that the endocrine, neurocognitive, and behavioral consequences of anabolic-androgenic steroid (AAS) use become more pronounced only after several repeated cycles of exposure, whereas effects during initial use are typically transient or subclinical (13, 17). To ensure clearly distinct exposure profiles, we applied a conservative two-year buffer between short-term and long-term categories, excluding intermediate users (1–3 years). This approach reduced misclassification risk and allowed us to examine contrasts between early-stage and chronic patterns of use, though we acknowledge that it may limit the generalizability of findings to intermediate users. Following this initial screening and group classification, participants were invited to attend a scheduled data collection session held at either Istanbul Nişantaşı University (European side) or Beykoz University (Asian side). These sessions took place in quiet laboratory settings and included administration of the Stroop Test as well as a comprehensive battery of psychological self-report measures. At the recruitment stage, a total of 436 individuals agreed to participate, comprising 125 classified as long-term users, 142 as short-term users, and 169 as non-users. However, not all invited participants attended the assessment sessions. Among the long-term users, 88 individuals completed the full testing protocol. One of these participants was subsequently excluded after it was discovered that he regularly used cannabis. Among the short-term users, 96 individuals completed the assessment session; one was excluded due to a documented diagnosis of ADHD in childhood. From the non-user group, 105 participants completed the assessment, but two were excluded after reporting current use of psychiatric medication (SSRIs). After accounting for attrition and exclusions, the final sample consisted of 87 long-term users, 95 short-term users, and 103 non-users. Participants provided self-reported details on the types of PEDs used, typical weekly dosages (where known), cycling patterns (on-/off-cycle duration), and post-cycle therapy (PCT) practices. These data were summarized descriptively to contextualize psychological findings, recognizing that self-report introduces some uncertainty in dosage accuracy. The participant selection and attrition process is summarized in Supplementary, which presents a CONSORT-style flow diagram detailing recruitment, screening, exclusion, and final group allocation.

### Inclusion and exclusion criteria

Participants were eligible if they were aged 18–55, identified as male or female, attended a gym at least three times weekly for the past six months, and met criteria for one of three groups: long-term PED users (over three years of use), short-term users (under one year), or non-users who had never used PEDs but reported body dissatisfaction and engaged in physical training. All had to be medically stable and able to complete assessments in Turkish.

Exclusion criteria included current use of psychiatric medications (e.g., SSRIs), history of chronic physical or neurological illness, or severe psychiatric conditions such as psychosis. Participants using substances like cannabis or medically prescribed PEDs (e.g., growth hormone, thyroid hormone, insulin) were also excluded, as were those with inconsistent or unreliable responses, especially regarding PED use or medical history.

### Measures

Multiple validated instruments were used to assess a range of psychological constructs. All scale instruments were administered in their Turkish validated versions, which have shown good psychometric properties in prior research.

Demographic and Background Information Form: A brief questionnaire developed for this study to collect personal data such as age, gender, education level, and gym/training history, as well as details of steroid/PED use (duration, frequency, types of substances) and reasons for use or non-use. This form also inquired about general health and any medication or substance use to confirm eligibility.

#### Beck depression inventory-II

The Beck Depression Inventory-II (BDI-II) is a 21-item self-report scale assessing depressive symptoms over the past two weeks, with items rated from 0 to 3 and total scores ranging from 0 to 63; higher scores indicate greater severity. The Turkish version, validated by Kapcı et al. (26), demonstrated excellent internal consistency (α = .90 nonclinical;.89 clinical) and strong test–retest reliability (r = .94). Factor analysis supported a two-factor structure in both clinical and nonclinical samples. Established cut-off scores are: 0–12 (minimal), 13–18 (mild), 19–28 (moderate), and 29–63 (severe). The BDI-II showed strong convergent and satisfactory discriminant validity.

#### Beck Anxiety Inventory

The Beck Anxiety Inventory (BAI) is a 21-item self-report scale developed to assess the severity of anxiety symptoms, particularly in clinical populations. Items reflect somatic, cognitive, and emotional aspects of anxiety and are rated on a 4-point Likert scale (0–3), yielding total scores from 0 to 63, with higher scores indicating greater anxiety. The Turkish version, validated by Ulusoy et al. (27), showed excellent internal consistency (α = .93) and acceptable test–retest reliability (r = .75). Factor analysis supported a two-factor structure—somatic and subjective anxiety—and the scale demonstrated strong discriminant validity, distinguishing anxiety from depression and other conditions, making it a reliable and culturally appropriate measure for Turkish adults.

#### Muscle Dysmorphic Disorder Inventory

The Muscle Dysmorphic Disorder Inventory (MDDI) is a 13-item self-report scale developed to assess muscle dysmorphia symptoms, with items rated on a 5-point Likert scale from “never” to “always.” Higher scores indicate greater symptom severity. It includes three subscales: Drive for Size (DFS), Appearance Intolerance (AI), and Functional Impairment (FI). The Turkish version, validated by Devrim and Bilgiç (28) in male bodybuilders, confirmed the original three-factor structure, explaining 50.2% of the variance, with factor loadings between 0.54 and 0.83. Internal consistency was acceptable (α = .66 total;.73 DFS;.66 AI;.60 FI), and two-week test–retest reliability was strong (ICC = .84). The scale showed good convergent validity with the EAT-40, FFMI, and body fat percentage, supporting its reliability and validity for Turkish bodybuilding populations.

#### General Self-Efficacy Scale

The General Self-Efficacy Scale (GSE), developed by Schwarzer and Jerusalem (1995), assesses overall confidence in handling various challenging situations. The 10-item version uses a 4-point Likert scale (1–4), with total scores ranging from 10 to 40; higher scores reflect greater perceived self-efficacy. The Turkish adaptation by Aypay (25), validated in a university sample (N = 693), revealed a two-factor structure—Effort and Persistence (6 items) and Ability and Confidence (4 items)—explaining 47% of the variance. Factor loadings ranged from.45 to.79. Internal consistency was strong (α = .83 overall;.79 and.63 for subscales), with high test–retest reliability over eight weeks (r = .80). The scale showed moderate criterion validity via correlations with the Rosenberg Self-Esteem Scale (r = .38) and Coping with Stress Scale—Problem Orientation (r = .40), supporting its reliability and validity in Turkish academic and clinical populations.

#### Multidimensional Scale of Perceived Social Support

The Multidimensional Scale of Perceived Social Support (MSPSS) is a 12-item self-report measure assessing perceived social support from Family, Friends, and a Significant Other, with each subscale comprising four items. Responses are rated on a 7-point Likert scale (1 = very strongly disagree to 7 = very strongly agree), with higher scores indicating greater perceived support. The Turkish adaptation by Başol (29) confirmed the original three-factor structure, explaining 77.2% of the variance. Internal consistency was excellent (α = .92 total;.90 Friends;.87 Family;.92 Significant Other), and split-half reliability was strong (r = .90). The MSPSS is a psychometrically robust tool widely used in both clinical and non-clinical Turkish populations.

#### Social Adaptation Self-Evaluation Scale

The Social Adaptation Self-Evaluation Scale (SASS) is a 21-item self-report tool assessing social functioning across four domains: work/productivity, interpersonal relationships, leisure/community engagement, and self-perception/autonomy. Items are rated on a 4-point scale (0–3), with total scores ranging from 0 to 60; higher scores indicate better functioning. Scores below 25 suggest impaired adaptation, while scores above 35 reflect normal functioning. The Turkish version, validated by Akkaya et al. (30) in patients with major depressive disorder and healthy controls, showed high internal consistency (α = .90 total;.87 clinical) and test–retest reliability (r = .77). It correlated strongly with the Global Assessment of Functioning (r = .62) and negatively with the Hamilton Depression Rating Scale (r = –.60). Factor analysis confirmed four components, with the first explaining 35.8% of the variance. The SASS also demonstrated sensitivity to treatment effects, supporting its validity and clinical utility in Turkish populations.

#### Stroop Color and Word Test (ÇAPA version)

The Stroop Color and Word Test (ÇAPA version) is a standardized neuropsychological tool used to assess executive functions, including selective attention, processing speed, and response inhibition. This version, developed by the Laboratory of Neuropsychology at Istanbul University based on Weintraub’s (2000) (31) adaptation, consists of three conditions: Stroop A (color naming), Stroop B (word reading), and Stroop C (interference), each containing 60 items arranged in six rows of ten. Stroop D, calculated as the time difference between Stroop C and B, reflects resistance to interference. In this study, the test was administered face-to-face in a distraction-free setting, using materials prepared according to Emek Savaş et al. (32), including proper color printing, PVC lamination, and structured layout. Participants were timed with a stopwatch, and both errors and corrections were recorded in real time. The ÇAPA version has demonstrated solid psychometric properties in Turkish samples, with test–retest reliability ranging from.64 to.88 across subtests and age groups, and internal consistency coefficients of α = .77 (standardized α = .86). Normative data are available for adults aged 18–83, stratified by age and education level.

### Data analysis

All statistical analyses were conducted using SPSS (version: 30), Python (version: 3.13.1) and the PROCESS macro (version: 4.0). Prior to hypothesis testing, data were screened for accuracy, normality, and missing values; no missing data were observed as all assessments were completed under supervision. Descriptive statistics were computed to summarize the sample characteristics and scale distributions. To examine group differences among long-term users, short-term users, and non-users, one-way analyses of variance (ANOVA) were performed on continuous outcome variables, including depression (BDI-II), anxiety (BAI), muscle dysmorphia (MDDI), cognitive performance (Stroop test), social functioning (SASS), self-efficacy (GSE), and perceived social support (MSPSS). Where significant main effects emerged, Bonferroni-adjusted *post hoc* t-tests or Tukey’s HSD were conducted. Planned independent-samples t-tests were also used to compare combined user groups versus non-users, and long-term versus short-term users on key outcomes. Effect sizes (Cohen’s d, partial η²) were calculated to assess the magnitude of observed differences. To explore continuous associations, multiple linear regression models were conducted within the user group to test whether PED use duration predicted outcomes such as depressive symptoms, anxiety, social functioning, or Stroop performance. All regression and ANCOVA models controlled for covariates including age, gender, and gym attendance frequency, given their potential influence on both PED use and psychological variables.

Mediation analyses were conducted using PROCESS Model 4 to test whether depression and anxiety mediated the relationship between PED use and social functioning (SASS). Bootstrap resampling (5,000 iterations) was used to estimate indirect effects with 95% confidence intervals. Moderation analyses (PROCESS Model 1) were used to test whether self-efficacy (GSE) or perceived social support (MSPSS) moderated the association between PED use and emotional outcomes (e.g., depression, anxiety) or body image disturbance (MDDI). Interaction terms were probed using simple slopes analysis at ±1 SD of the moderator. All analyses were two-tailed with a significance level of p <.05. Assumptions of normality and homogeneity of variance were confirmed for all parametric tests. Where assumptions were marginally violated, sensitivity checks were conducted using appropriate non-parametric alternatives. All analyses were first conducted using unadjusted models to present raw group-level comparisons. Subsequently, covariate-adjusted analyses (ANCOVAs and hierarchical regressions) were performed controlling for age, gender, and weekly training frequency to test the robustness of group effects. As these adjustments did not alter the direction or significance of results, descriptive tables and figures present unadjusted means for interpretability, while the text notes that covariate-controlled results yielded comparable outcomes. In all mediation and moderation models, categorical predictors were dummy-coded using the PROCESS macro’s default structure (0 = user, 1 = non-user). Accordingly, negative unstandardized coefficients represent higher scores among users relative to non-users, while positive coefficients represent higher scores among non-users.

### Power analysis

A *post hoc* power analysis using G*Power 3.1 confirmed that the final sample (N = 285) was sufficient for detecting medium effect sizes across all planned analyses. For one-way ANOVA (f = 0.25, α = .05, power = .80), the required sample size is 159. For multiple regression (f² = 0.15, five predictors), the required sample is 92. Both thresholds were exceeded. The sample also meets recommended guidelines (N ≥ 150–200) for mediation and moderation analyses using the PROCESS macro with bootstrapping.

## Results

### Participant characteristics

A total of N = 285 participants were included in the analyses: 87 long-term PED users, 95 short-term users, and 103 non-users. The sample was predominantly male (76.8%), with group differences observed in age, gender, and training frequency. As expected, long-term users were older on average (M = 30.4 years) than short-term users (M = 26.1) and non-users (M = 25.3), reflecting the ≥3-year use criterion. Training frequency also differed significantly, with long-term users training most frequently (M = 5.5 days/week), and non-users the least (M = 4.1 days/week). The proportion of female participants was highest among non-users (42%), consistent with the higher prevalence of PED use among men. No significant group differences were found for education level or smoking status.

**Figure 1:**
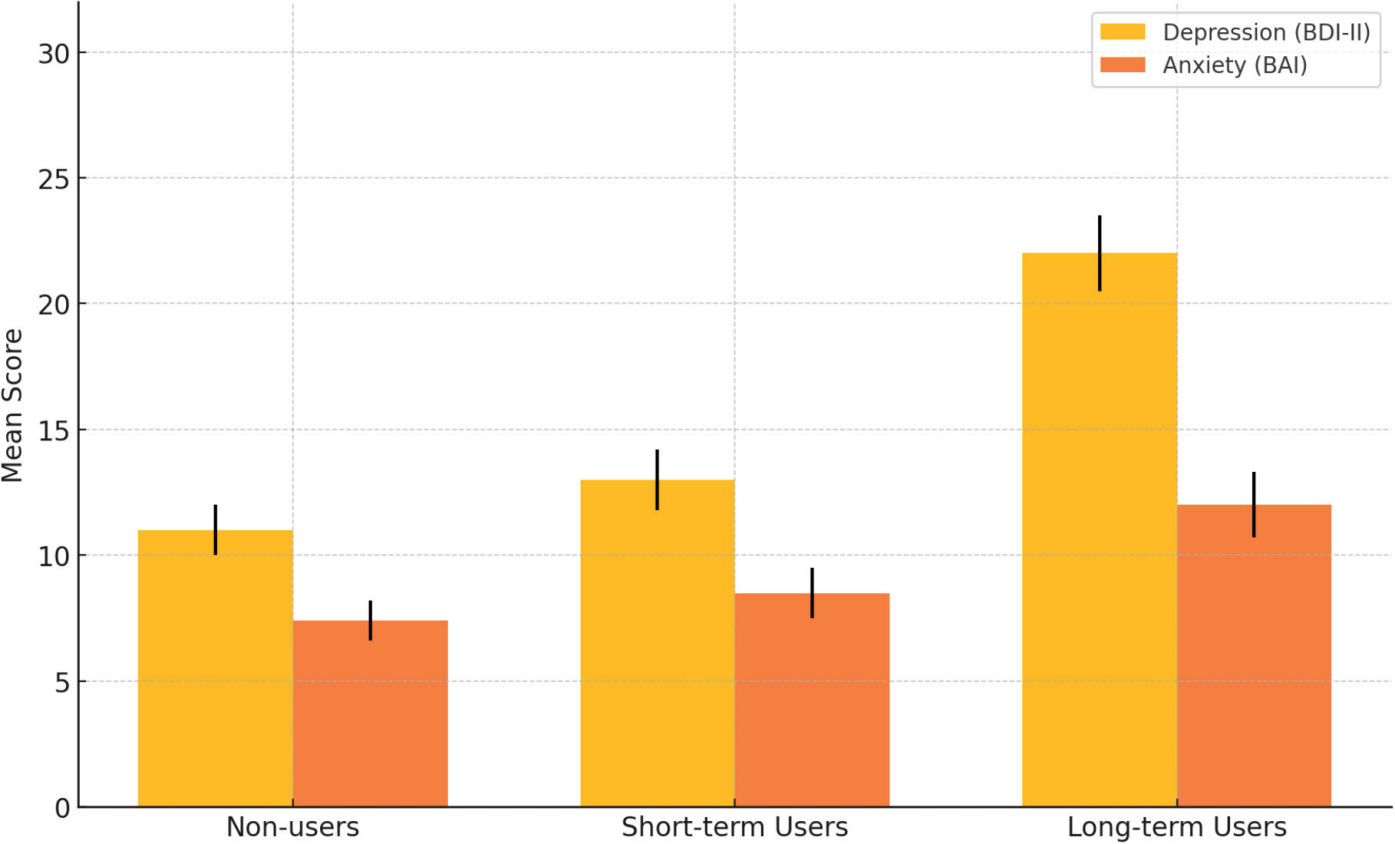
Psychological symptoms by PED use group.

**Figure 2:**
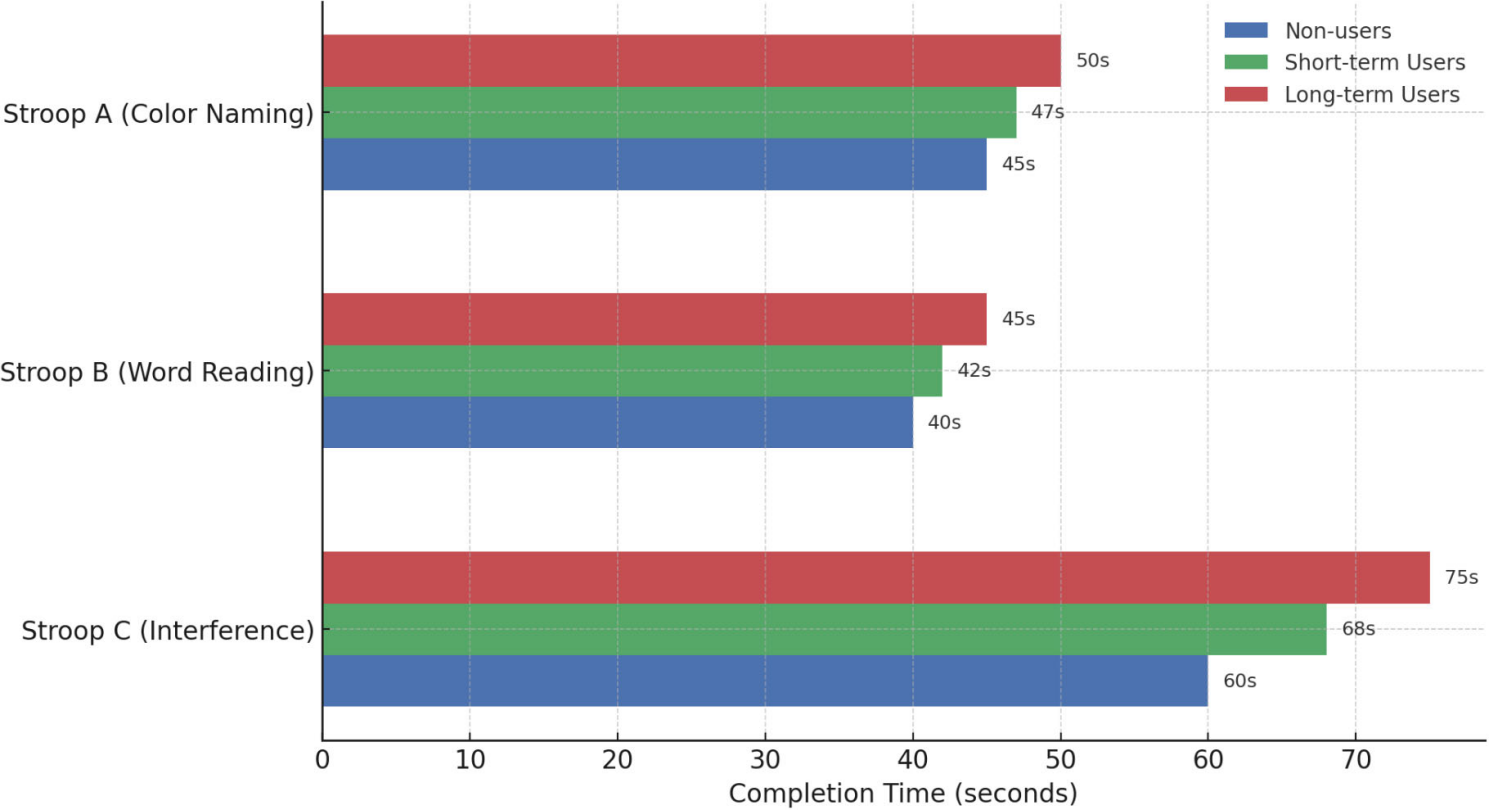
Stroop A, B, C times by group.

As can be seen in **Table 1A**, there were no significant baseline differences in depression or anxiety scores by gender (independent t tests, p >.10), so subsequent analyses were collapsed across gender. All continuous outcome variables met assumptions of normality and homogeneity of variance. Covariate-adjusted analyses (ANCOVAs controlling for age, gender, and training frequency) yielded very similar results to the unadjusted analyses. Covariate-adjusted ANCOVA models controlling for age, gender, and training frequency yielded the same significance pattern as the unadjusted analyses (all p <.05), confirming the robustness of the group effects presented in **Tables 2**–**5** and **Figures 1**–**2**.

**Table 1A:** Sample characteristics by group.

Variable	Long-term users (n = 87)	Short-term users (n = 95)	Non-users (n = 103)	F or χ²	P
Age (years)	30.4 (± 7.8)	26.1 (± 6.5)	25.3 (± 6.9)	15.27	<.001 *
Male (%)	92.0%	83.2%	57.3%	36.45^a^	<.001 *
College Education (%)	48.3%	50.5%	53.4%	0.79^a^	.674
Current Smoker (%)	21.8%	18.9%	24.3%	1.02^a^	.600
Training Frequency (days/week)	5.5 (± 1.2)	4.8 (± 1.4)	4.1 (± 1.3)	29.11	<.001 *

Values are Mean (± SD) for continuous variables and percentages for categorical variables. p <.05 marked with *; ^a^Chi-square test.

**Table 1B:** Self-reported PED use patterns among short- and long-term users.

Substance/Pattern	Short-term users (n = 95) %	Long-term users (n = 87) %	Notes
Primary Injectable AAS			
Testosterone esters (enanthate/cypionate)	54	83	Most common base compound
Nandrolone decanoate (Deca-Durabolin)	22	48	Often stacked with testosterone
Trenbolone (acetate/enanthate)	6	29	Predominantly long-term users
Oral AAS			
Stanozolol (Winstrol)	33	47	Used mainly during cutting cycles
Methandienone (Dianabol)	27	39	Frequent starter compound
Oxandrolone (Anavar)	14	21	Used by both genders for mild cycles
Other PEDs/Adjuncts			
Human growth hormone (HGH)	5	19	Mostly cosmetic/anti-aging intent
Clenbuterol	11	24	Used for fat-loss phases
Cycle Pattern			
Continuous year-round use	12	38	Typical of dependence patterns
On/off cycles (8–12 weeks)	68	49	Standard gym-based model
Irregular/unclear	20	13	Unverified routines
Post-Cycle Therapy (PCT)			
Yes (SERMs/AI reported)	29	55	Tamoxifen, clomiphene most cited
No/unsure	71	45	Often self-reported “natural recovery”

Percentages reflect valid responses within user subgroups. Dosage values were not analyzed statistically due to high variance and incomplete recall.

**Table 2:** Descriptive statistics for psychological and behavioral variables by group.

Variable	Scale range	Non-usersM (± SD)	Short-term usersM (± SD)	Long-term usersM (± SD)	F (2, 282)	P
BDI-II (Depression)	0 – 63	11 (7)^a^	13 (8)^a^	22 (10)^b^;	42.2	<.001 *
BAI (Anxiety)	0 – 63	7.4 (6)^a^	8.5 (7)^a^	12 (8)^b^;	10.8	<.001 *
GSE (Self-Efficacy)	10 – 40	29.8 (5.0)^a^	28.3 (5.2)^a^	29.6 (5.1)^a^	2.79	.063
MSPSS (Social Support)	1 – 7 (mean/item)	5.84 (0.9)^a^	5.38 (1.0)^b^;	5.15 (1.0)^b^;	13.2	<.001 *
MDDI Total (Muscle Dysmorphia)	13 – 65	30 (10)^a^	38 (11)^b^;	43 (12)^b^;	37.4	<.001 *
Drive for Size (DFS)	4 – 20	9 (4)^a^	15 (5)^b^;	16 (6)^b^;	33.2	<.001 *
Appearance Intolerance (AI)	4 – 20	10 (4)^a^	12 (5)^b^;	13 (6)^b^;	6.1	.003 *
Functional Impairment (FI)	4 – 20	11 (4)^a^	12 (5)^ab^;	14 (5)^b^;	4.5	.012 *
Stroop A (Color Naming)	Time (sec)	45 (9)^a^	47 (10)^a^	50 (11)^a^	2.40	.090
Stroop B (Word Reading)	Time (sec)	40 (8)^a^	42 (9)^a^	45 (10)^b^;	3.10	.047 *
Stroop C (Interference)	Time (sec)	60 (12)^a^	68 (14)^b^;	75 (16)^c^	12.50	<.001 *
SASS (Social Functioning)	0 – 60	40.5 (9)^a^	35.4 (9)^b^;	30.1 (8)^c^	34.8	<.001 *

Means that do not share a common superscript differ significantly at *p* <.05 (Tukey’s HSD *post-hoc* tests). Higher BDI-II, BAI, MDDI, and Stroop times indicate greater symptom severity or slower performance; higher GSE, MSPSS, and SASS scores indicate better functioning or greater psychosocial resources. Group means are unadjusted for covariates; parallel ANCOVA models controlling for age, gender, and training frequency produced consistent significance patterns. * p <.05.

**Table 3:** Stroop test performance by condition and group.

Condition	Non-users (M ± SD)	Short-term users (M ± SD)	Long-term users (M ± SD)	F	p
Stroop A(Color Naming)	45 ± 9	47 ± 10	50 ± 11	2.40	.090
Stroop B(Word Reading)	40 ± 8	42 ± 9	45 ± 10	3.10	.047 *
Stroop C(Interference)	60 ± 12	68 ± 14	75 ± 16	12.50	<.001 *

Higher completion times indicate slower cognitive performance (greater difficulty with the task). p <.05 marked with *. Group means are unadjusted for covariates. Parallel ANCOVA models controlling for age, gender, and training frequency produced consistent significance patterns.

**Table 4:** Group differences on MDDI muscle dysmorphia subscale scores.

MDDI subscale	Non-users (n = 103)	Short-term users (n = 95)	Long-term users (n = 87)	F	P
Drive for Size (DFS)	9 ± 4	15 ± 5	16 ± 6	33.2	<.001 *
Appearance Intolerance (AI)	10 ± 4	12 ± 5	13 ± 6	6.1	.003 *
Functional Impairment (FI)	11 ± 4	12 ± 5	14 ± 5	4.5	.012 *

Higher scores reflect greater muscle dysmorphic symptoms.

MDDI, Muscle Dysmorphia Disorder Inventory; DFS, Drive for Size; AI, Appearance Intolerance; FI, Functional Impairment. p <.05 marked with *. Group means are unadjusted for covariates. Parallel ANCOVA models controlling for age, gender, and training frequency produced consistent significance patterns.

**Table 5:** Group differences in self-efficacy, perceived social support, and social functioning.

Measure	Non-users (M ± SD)	Short-term users (M ± SD)	Long-term users (M ± SD)	F	p	η²
Self-Efficacy (GSE)	29.8 ± 5.0	28.3 ± 5.2	29.6 ± 5.1	2.79	.063	—
Perceived Social Support	5.84 ± 0.9	5.38 ± 1.0	5.15 ± 1.0	13.2	<.001 *	.09
Social Functioning (SASS)	40.5 ± 9.0	35.4 ± 9.0	30.1 ± 8.0	34.8	<.001 *	.20

p <.05 marked with *.

Higher GSE, MSPSS, and SASS scores reflect better functioning or greater psychosocial resources.

**Table 1B** presents the distribution of reported PED types, cycling characteristics, and post-cycle therapy practices among long- and short-term users. Testosterone esters (e.g., enanthate, cypionate) and nandrolone were the most frequently reported compounds, followed by oral agents such as stanozolol and methandienone. The majority of long-term users (71%) reported multi-compound “stacking,” compared to 28% of short-term users. About two-thirds of users employed cyclical on/off patterns averaging 8–12 weeks per cycle, and 42% reported at least occasional PCT use (commonly tamoxifen or clomiphene). Only 18% could provide approximate dosage information; thus, values are presented as typical ranges rather than precise milligrams.

### Descriptive statistics for psychological and behavioral measures

**Table 2** provides descriptive statistics (means and standard deviations) for all primary psychological, cognitive, and social variables across the three PED use groups.

Descriptive statistics for all primary outcome variables are presented in **Table 2**. As shown, long-term PED users generally reported higher depression, anxiety, and muscle dysmorphia scores and exhibited slower Stroop performance and lower social functioning than both short-term users and non-users. Short-term users showed intermediate values on most measures. Group differences in self-efficacy were minimal, while perceived social support and social functioning declined with increased PED use duration. All mean scores fell within valid ranges for their respective scales.

### Group comparisons on psychological measures

Consistent with H1, long-term PED users reported the highest depression and anxiety levels, while short-term users’ scores were closer to non-users. A one-way ANOVA on BDI-II scores showed a significant group effect, F(2,282) = 42.2, p <.001, η² = .23. Long-term users (M = 22, SD = 10) scored significantly higher than short-term users (M = 13, SD = 8) and non-users (M = 11, SD = 7; both p <.001). Short-term users did not differ significantly from non-users (p = .21). A similar pattern was found for BAI scores, F(2,282) = 10.8, p <.001, η² = .07: long-term users (M = 12, SD = 8) scored higher than non-users (M = 7.4, SD = 6, p <.001) and short-term users (M = 8.5, SD = 7, p = .002), with no difference between short-term users and non-users (p = .51).

Bar graph in **Figures 1** displays mean scores (± 1 SE) on the Beck Depression Inventory-II (BDI-II) and Beck Anxiety Inventory (BAI) for non-users, short-term PED users, and long-term users. Long-term users reported the highest levels of both depression and anxiety, with statistically significant differences compared to the other groups. Short-term users’ scores were modestly elevated for anxiety but did not differ significantly from non-users on either scale. This pattern suggests that chronic PED use is associated with pronounced emotional distress, while short-term use shows weaker and less consistent effects.

### Cognitive performance

Consistent with H2, long-term PED users showed significantly poorer performance on the Stroop interference task. A one-way ANOVA revealed a group effect, F(2,282) = 12.8, p <.001. Long-term users had the longest interference times (M = 75s), indicating slower cognitive processing, compared to short-term users (M = 68s) and non-users (M = 60s). Higher times reflect worse inhibition. *Post hoc* tests showed long-term users performed significantly worse than both non-users and short-term users (p <.001). Short-term users did not differ significantly from non-users (p = .08), suggesting minimal impairment. These findings support H2, indicating that cognitive slowing is more pronounced with extended PED use.

As summarized numerically in **Table 3**, **Figure 2** offers a graphical depiction of group performance across Stroop conditions, highlighting a dose–response pattern in cognitive inhibition deficits among PED users.

In **Figure 2**, long-term PED users showed the slowest performance across all Stroop conditions, especially in the interference task (C), supporting H2. Completion times increased with PED use duration: non-users were fastest, short-term users intermediate, and long-term users slowest. This pattern suggests that extended steroid use may impair cognitive processing, particularly executive function.

### Muscle dysmorphia

Muscle dysmorphia symptoms varied significantly across groups, F(2,282) = 37.4, p <.001, η² = .21. Long-term users reported the highest overall MDDI scores (M = 43, SD = 12), followed by short-term users (M = 38, SD = 11), and non-users (M = 30, SD = 10). Both user groups scored significantly higher than non-users (p <.001), while the difference between long- and short-term users was marginal (p = .07). Subscale analyses revealed that PED users scored higher than non-users across all three domains: Drive for Size, Appearance Intolerance, and Functional Impairment, with long-term users consistently showing the greatest symptom burden. As presented in **Table 4**, these findings suggest that even limited PED use is associated with elevated body image disturbance, and symptoms may intensify with prolonged use.

### Self-efficacy and social resources

General self-efficacy scores did not differ significantly across groups, F(2,282) = 2.79, *p* = .063. Although non-users showed slightly higher mean scores (M = 29.8) than short-term (M = 28.3) and long-term users (M = 29.6), these differences were not statistically reliable. This suggests that any protective effects of self-efficacy observed in moderation analyses are unlikely due to baseline group differences.

In contrast, perceived social support varied significantly by group, F(2,282) = 13.2, *p* <.001, η² = .09. Non-users reported the highest support levels, followed by short-term users, with long-term users reporting the lowest. Social functioning followed a similar pattern, F(2,282) = 34.8, *p* <.001, η² = .20, with a clear gradient from non-users (highest functioning) to long-term users (lowest functioning). As shown in **Table 4**, these results underscore the importance of social resources in relation to PED-related psychological outcomes.

### Planned contrasts (targeted group comparisons)

Planned contrasts addressing RQ1 showed that PED users, regardless of duration, exhibited significantly worse outcomes than non-users across multiple domains, including depression, anxiety, muscle dysmorphia, social functioning, and perceived social support. However, self-efficacy did not differ significantly between groups. Comparisons between long-term and short-term users revealed that psychological impairment was more pronounced with prolonged use—particularly for depression, cognitive performance (Stroop), and social functioning—whereas anxiety, social support, and dysmorphia did not differ significantly. These results, summarized in **Table 6**, suggest a cumulative psychological cost associated with long-term PED use, although certain vulnerabilities may emerge even in early phases of use.

**Table 6:** Planned contrasts comparing PED users *vs*. non-users and long-term *vs*. short-term users.

Outcome measure	PED users *vs*. non-users			Long-term *vs*. short-term		
	*t*	*p*	*d*	*t*	*p*	*d*
Depression (BDI-II)	8.52	<.001 *	1.01	4.11	<.001 *	0.61
Anxiety (BAI)	3.55	<.001 *	0.42	1.53	.130	—
Muscle Dysmorphia (MDDI)	7.98	<.001 *	0.95	1.53	.130	—
Social Functioning (SASS)	–7.93	<.001 *	0.94	–2.02	.045 *	—
Social Support (MSPSS)	–3.57	<.001 *	—	–1.56	.120	—
Self-Efficacy (GSE)	–1.65	.100	—	—	—	—
Stroop Performance	—	—	—	–2.45	.015 *	0.36

p <.05 marked with *.

Cohen’s d not reported where unavailable or non-calculated.

### Regression analyses: predictors of depression and anxiety among PED users (RQ2)

Regression Analyses: Predictors of Depression and Anxiety Among PED Users (RQ2).

To explore RQ2, multiple regression analyses were conducted within the PED-using sample (n = 182). Longer PED use duration predicted higher depression (β = .32, p <.001), but not anxiety (p = .10), and was also associated with poorer social functioning and slower Stroop performance. Bivariate correlations showed that muscle dysmorphia and low social support were strongly associated with both depression and anxiety, while self-efficacy had weaker, non-significant associations. In multivariate models, muscle dysmorphia and low perceived social support emerged as robust predictors of both outcomes, whereas self-efficacy was not significant. Summarized in **Table 7**, these findings suggest that emotional distress in PED users is more closely linked to body image disturbance and social environment than to self-belief alone.

**Table 7:** Multiple regression results predicting depression and anxiety among PED users.

Predictor	Depression (BDI-II)B (SE)	P	Anxiety (BAI)B (SE)	P
Muscle Dysmorphia (MDDI)	0.50 (0.08)	<.001 *	0.35 (0.08)	<.001 *
Social Support (MSPSS)	–2.00 (0.60)	.002 *	–1.50 (0.50)	.005 *
Self-Efficacy (GSE)	–0.30 (0.25)	.120	–0.20 (0.15)	.180

Model R² ≈ 0.45 for depression and 0.37 for anxiety (both p <.001). Unstandardized coefficients are shown. Negative B values indicate that higher scores on the predictor correspond to lower symptom severity. Covariates (age, gender, training frequency) were included in Step 1 of each model. p <.05 marked with *.

### Mediation analysis: emotional distress as a mediator of social dysfunction (H3)

**Figure 3:**
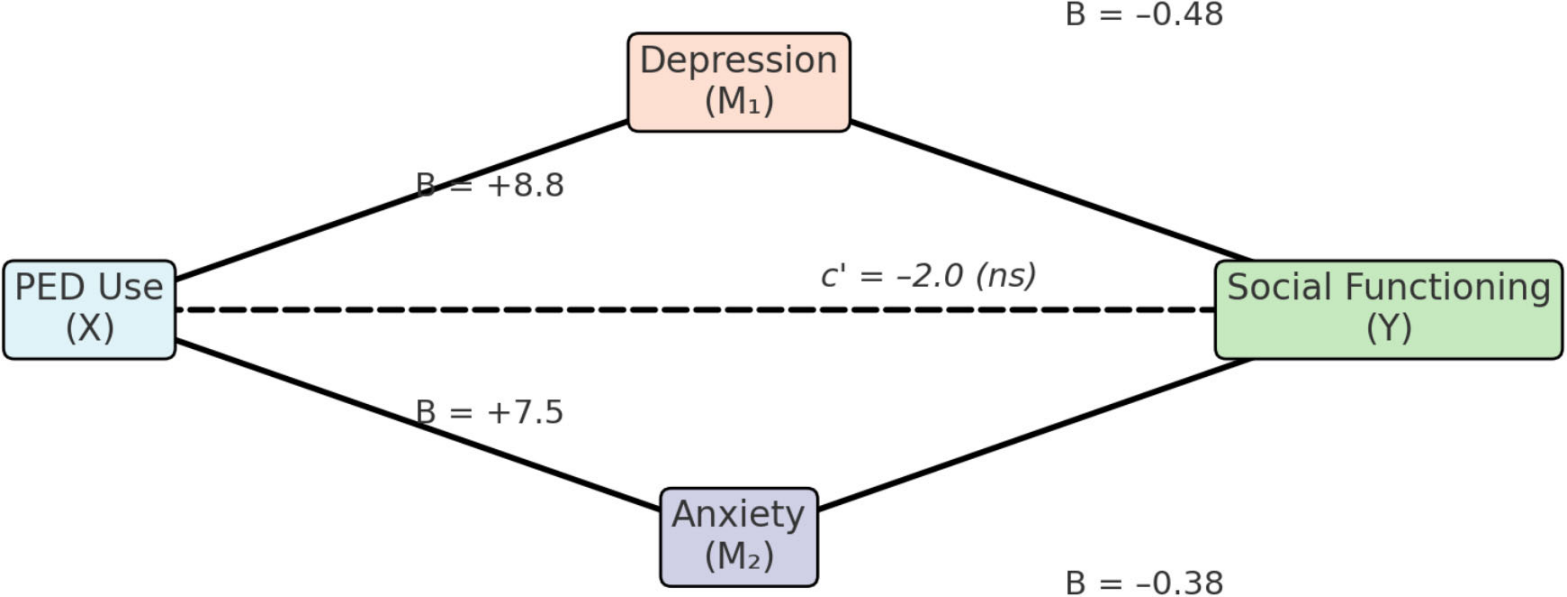
Emotional distress as a mediator of social dysfunction.

To test H3, a parallel multiple mediation analysis (PROCESS Model 4) examined whether depression and anxiety mediated the link between PED use (user *vs*. non-user) and social functioning (SASS scores). PED use had a significant total effect on social functioning (B = –8.7, p <.001), with users reporting lower functioning. PED use also significantly predicted both depression and anxiety (p <.001), and in turn, both were associated with poorer social functioning (p <.001). As visualized in **Figures 3**, these results support partial mediation, indicating that emotional distress contributes to the negative social outcomes associated with PED use. The negative path coefficients from PED Use (X) to the mediators (depression and anxiety) reflect the dummy-coding scheme (0 = user, 1 = non-user), indicating that users reported higher levels of emotional distress. Thus, although the coefficients appear negative, they correctly represent the expected direction of effect, with greater depression and anxiety among PED users.

**Figure 3**. Parallel mediation model testing depression (M_1_) and anxiety (M_2_) as mediators of the association between PED use (X: 0 = user, 1 = non-user) and social functioning (Y: SASS score). Negative path coefficients from X to the mediators indicate higher depression and anxiety among PED users. Unstandardized coefficients are displayed. Indirect effects were significant for both mediators (p <.05), confirming partial mediation. The direct effect of PED use on social functioning (c’ path, shown in parentheses) was reduced and no longer statistically significant when accounting for depression and anxiety (p = .08), suggesting that the association between PED use and social impairment was largely – though not entirely – explained by elevated emotional distress.

Bootstrap analysis (5,000 resamples) confirmed significant indirect effects of PED use on social functioning through both depression (B = –4.21, 95% CI [–6.08, –2.60]) and anxiety (B = –1.56, 95% CI [–2.79, –0.58]), with both confidence intervals excluding zero. Together, these mediators accounted for approximately 60–65% of the total effect. The direct effect of PED use on social functioning dropped to B ≈ –2.0 and became non-significant (p = .11; bias-corrected p = .08), indicating partial mediation. These findings support H3: the social difficulties observed in PED users were largely explained by elevated depression and anxiety, though a small, non-significant direct effect remained.

### Moderation analyses: protective factors (H4 & H5)

To test H4, we examined whether self-efficacy (GSE) moderated the relationship between PED use and depression. A moderation model (PROCESS Model 1), controlling for covariates, showed a significant interaction between PED use and self-efficacy (B = –0.50, SE = 0.22, t = –2.27, p = .024, ΔR² ≈.02). Simple slopes analysis revealed that at low self-efficacy (–1 SD), PED use predicted significantly higher depression (B = +8.8, p <.001), while at high self-efficacy (+1 SD), the effect was non-significant (B = +0.9, p = .68). These results support H4, indicating that high self-efficacy buffers the depressive impact of PED use.

**Figure 4:**
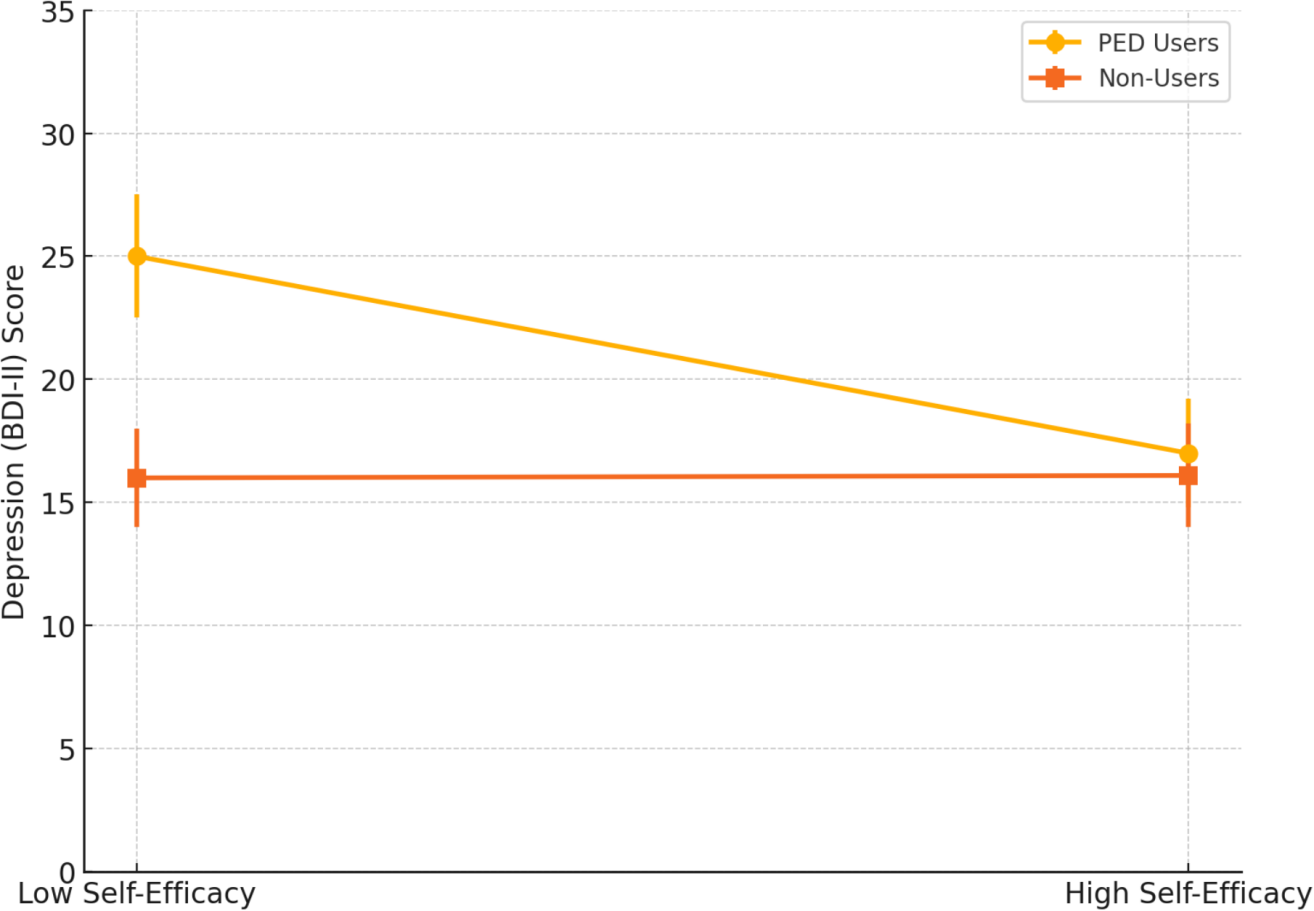
Moderation of PED use-depression relationship by self-efficacy.

**Figure 4** shows moderation of the PED use–depression relationship by self-efficacy (H4). The graph plots mean depression (BDI-II) scores for PED users *vs*. non-users at low (–1 SD) and high (+1 SD) self-efficacy levels. Error bars indicate ±1 SE. Among individuals with low self-efficacy, PED users show significantly higher depression than non-users; by contrast, at high self-efficacy, the depression difference between users and non-users is minimal. This suggests that strong self-efficacy attenuates the depressive impact of steroid use, consistent with a stress-buffering effect.

Following the self-efficacy moderation results presented in **Figure 4**, we conducted additional moderation analyses testing whether perceived social support also buffered against depression and anxiety among PED users. We tested whether perceived social support (MSPSS) moderated the relationship between PED use and emotional well-being. In predicting depression (BDI-II), the interaction between PED use and social support was significant (B = –3.21, SE = 1.45, p = .028). Simple slope analysis showed that PED users reported much higher depression at low support (–1 SD; B = +7.5, p <.001), but this difference was negligible at high support (+1 SD; B = +1.2, p = .56), indicating a buffering effect. A similar pattern emerged for anxiety (BAI), with a significant interaction (p = .037), suggesting that high perceived support reduced anxiety differences between users and non-users. No moderation effects were found for muscle dysmorphia. Overall, high social support protected against emotional distress linked to PED use, consistent with H5.

**Table 8** summarizes moderation analyses examining whether self-efficacy and social support buffer the psychological impact of PED use. Significant interactions were found for depression: both higher self-efficacy (p = .024) and greater social support (p = .028) attenuated depressive symptoms among users. Social support also moderated anxiety (p = .037), while effects for self-efficacy on anxiety and for both moderators on muscle dysmorphia were non-significant. These findings highlight the protective role of psychosocial resources, particularly against emotional distress.

**Table 8:** Interaction effects of PED use and psychosocial moderators on psychological outcomes.

Outcome (DV)	Moderator	Interaction B	SE	P
Depression (BDI-II)	Self-Efficacy (GSE)	–0.50	0.22	.024 *
Anxiety (BAI)	Self-Efficacy (GSE)	–0.35	0.25	.090
Depression (BDI-II)	Social Support (MSPSS)	–3.21	1.45	.028 *
Anxiety (BAI)	Social Support (MSPSS)	–2.50	1.20	.037 *
Muscle Dysmorphia (MDDI)	Self-Efficacy (GSE)	–0.55	0.50	.290
Muscle Dysmorphia (MDDI)	Social Support (MSPSS)	–1.00	1.10	.340

Unstandardized interaction coefficients from PROCESS Model 1 (PED Use × Moderator). Significant interactions (p <.05) indicate a buffering or exacerbating effect of the moderator on the relationship between PED use and the dependent variable.

*statistically significant at the conventional p <.

## Discussion

The present study contributes new evidence in two particularly novel areas: (a) a dose–response pattern indicating that longer durations of PED use correspond to greater emotional, cognitive, and social difficulties, and (b) the identification of psychosocial resources—self-efficacy and perceived social support—as key protective moderators of these associations. We examined the emotional, cognitive, and social correlates of performance-enhancing drug (PED) use among gym-going men and women. Overall, the findings broadly supported our hypotheses, with notable distinctions based on duration of use. Long-term anabolic steroid (AAS) users reported significantly higher levels of depression and anxiety (H1), poorer executive functioning (H2), and reduced social functioning compared to non-users. Mediation analysis (H3) suggested that emotional distress largely accounted for their social impairment. In contrast, short-term users showed fewer psychological deficits and more closely resembled non-users across most outcomes, though some elevation in muscle dysmorphia was observed. Moderation analyses further revealed that high self-efficacy (H4) and strong perceived social support (H5) buffered users against depression and anxiety. These findings highlight the differential impact of PED use duration (RQ1) and the critical role of personal and social resources in shaping mental health outcomes among users (RQ2).

A key finding of this study is the strong association between long-term PED use and elevated depression and anxiety symptoms (33, 34). This finding aligns with prior evidence linking sustained anabolic-androgenic steroid (AAS) exposure to higher psychological distress. In our sample, long-term users exhibited BDI-II scores within the moderate clinical range, significantly higher than both short-term users and non-users, consistent with previous observations (35). While short-term steroid use may coincide with transient mood elevation, extended or post-cycle phases often correspond to increased depressive symptoms, potentially related to hormonal suppression (36, 37). Many participants categorized as long-term users reported being off-cycle at the time of assessment, which may partially explain their higher depression levels, consistent with self-reports of mood “crashes” during withdrawal (38). Elevated anxiety in this group may likewise reflect neuroendocrine or neurotransmitter dysregulation (e.g., GABAergic or serotonergic pathways).

In contrast, short-term users did not significantly differ from non-users on mood measures, suggesting that adverse emotional effects may develop over time. Some may have been on-cycle during data collection, potentially masking depressive symptoms. Importantly, not all long-term users reported high distress (39). A subset showed normal-range depression scores, often accompanied by high self-efficacy and strong social support—factors shown to buffer emotional effects (H4, H5; 40). Similarly, some non-users reported elevated distress, reinforcing that PED use is not the sole predictor of mood problems. These findings highlight the complexity and individual variability in emotional outcomes and underscore the need for mental health screening and support among long-term AAS users, especially during withdrawal periods.

Long-term PED users in our study showed significantly worse performance on the Stroop task, indicating deficits in attention, processing speed, and inhibitory control. This aligns with prior research reporting executive and memory impairments among chronic AAS users (41, 42). Neuroimaging studies support these findings, linking prolonged steroid use to frontal cortical thinning, reduced gray matter, and hippocampal dysfunction (43–45). These neural changes may underlie slowed processing and poor impulse control, with potential consequences for decision-making and daily functioning. Short-term users, by contrast, showed minimal cognitive impairment, suggesting that serious deficits likely require prolonged exposure (46). A selection effect may also be at play—those who experience early cognitive side effects may stop using (47). Overall, the results caution that PEDs may harm not just the body, but also the brain, and highlight the need for longitudinal research on neurocognitive outcomes in aging users.

Long-term PED users reported significantly poorer social functioning (48, 49), with the lowest scores on the SASS scale, indicating difficulties in relationships, work, and community engagement (50). These findings align with qualitative accounts describing social withdrawal and interpersonal conflict among steroid users (51). Behavioral changes linked to AAS—such as aggression (52), irritability (53), and mood swings (54)—likely contribute to strained relationships. Some studies also associate heavy steroid use with personality changes (e.g., increased anger, psychopathic traits), which can further impair social integration.

Mediation analysis revealed that emotional distress (depression and anxiety) largely accounted for users’ poor social outcomes (55). When controlling for mood, the direct effect of PED use on social functioning was no longer significant, suggesting that emotional symptoms are the primary drivers of interpersonal problems. This fits with well-established models where depression disrupts social motivation and role fulfillment (56). However, causality may be bidirectional. Some individuals may begin using steroids due to pre-existing social deficits or low self-efficacy, only to experience worsening relationships due to steroid-induced mood and behavior changes—a feedback loop documented in qualitative studies. Additional contributors include deficits in empathy and social cognition. For example, AAS users have shown reduced accuracy in recognizing emotional facial expressions (57), potentially leading to social missteps. Moreover, stigma may push users to conceal their behavior, limiting support from non-using peers and fostering insular, hyper-competitive gym subcultures. Our long-term users also reported significantly lower perceived social support.

Both short- and long-term PED users in our study reported significantly elevated muscle dysmorphia (MD) symptoms compared to non-users, highlighting the central role of body-image disturbance in steroid use (58). MD was also one of the strongest correlates of depression and anxiety, suggesting that these concerns are not superficial but clinically significant (59). Many users likely begin PED use in response to pre-existing body dissatisfaction and low self-efficacy (60). Our findings support this: even short-term users showed higher MDDI scores than non-users, despite limited steroid exposure. There is also evidence that steroid use itself exacerbates MD symptoms. Users may continuously shift their muscular ideals upward, perpetuating dissatisfaction (61). Long-term users in our study had the highest MD scores, pointing to a potential feedback loop: steroid use intended to resolve insecurities may ultimately deepen them. The strong associations between MD and emotional distress underscore the need for integrated treatment approaches. Addressing body-image beliefs—through interventions like cognitive-behavioral therapy—may be essential to alleviate depression and anxiety in this population.

Regarding self-efficacy, we found no large baseline group differences in General Self-Efficacy (GSE) scores. However, moderation analyses showed that higher self-efficacy buffered the association between PED use and depressive symptoms. Individuals with stronger beliefs in their ability to manage challenges reported significantly less emotional distress, consistent with stress-buffering models. Those whose sense of personal competence extended beyond physical appearance appeared less affected by steroid-related fluctuations (e.g., physique changes, mood variability), whereas those with more limited confidence in coping showed greater vulnerability. These findings suggest that strengthening perceived self-efficacy—rather than global self-efficacy—could be a key target for psychological support and prevention efforts among PED users (62). This interpretation is consistent with recent theoretical perspectives emphasizing motivational and self-regulatory mechanisms in athletic and performance contexts, where self-efficacy and intrinsic motivation play central roles in sustaining or mitigating performance-enhancing behaviors (63).

Our findings underscore the critical buffering role of perceived social support in the psychological well-being of PED users. Steroid users with strong support from family, friends, or partners reported emotional health comparable to non-users, whereas those with low support exhibited markedly higher depression and anxiety (64). This pattern is consistent with robust evidence in health psychology showing that social support mitigates stress and protects against mood disorders. Supportive relationships may offer emotional reassurance, practical coping strategies, and a sense of belonging that counteract the isolation and stigma often associated with PED use. Conversely, users reporting low perceived support exhibited significantly greater emotional distress. These findings suggest that social support not only mitigates the psychological burden of long-term PED use but may also protect against the onset of depressive and anxious symptoms. Clinically, promoting supportive social networks and peer-based interventions—particularly within gym communities—could enhance resilience, reduce secrecy, and protect against emotional decline (65). Evidence from research on female AAS users similarly indicates that those with stable support systems experience fewer psychological issues, reinforcing that social context is a critical protective factor against severe outcomes such as mood deterioration or suicidal ideation during withdrawal phases.

Our findings underscore that duration of PED use is a critical determinant of psychological outcomes. Short-term users (<1 year) generally showed less impairment than long-term users (>3 years), with significantly lower depression and anxiety, better cognitive performance, and fewer social difficulties—though both groups exhibited elevated muscle dysmorphia. These differences likely reflect both exposure and selection effects. Longer use entails greater physiological disruption (e.g., to the HPG axis and neurotransmitter systems), higher doses, multiple cycles, and increased risk of dependence—all of which heighten psychiatric risk. In contrast, therapeutic steroid use at lower doses rarely yields such consequences, reinforcing the role of chronic high exposure. Selection effects may also shape these patterns. Individuals who tolerate steroids without severe side effects may continue long-term, while those with adverse reactions may stop early. However, the fact that long-term users in our study showed more psychological issues—even with potential survivor bias—suggests cumulative harm over time. Correlations between use duration and both depression (positive) and cognitive performance (negative) support this. Conversely, psychological vulnerability may precede and sustain PED use. Those with pre-existing mood issues or body dissatisfaction may be more likely to start and persist. This may explain elevated MD even in short-term users and higher psychopathology in long-term users.

Together, these findings suggest that psychological risk associated with prolonged PED use may be partly offset by personal and social resilience factors, highlighting potential targets for prevention and intervention efforts.

### Limitations and future directions

This study’s cross-sectional design limits causal inference. While we interpret PED use as contributing to psychological changes, it is possible that pre-existing issues (e.g., depression, muscle dysmorphia) increase the likelihood of PED use. Longitudinal studies tracking individuals before, during, and after use are needed to clarify directionality. Self-selection and reporting bias are also concerns. Participants were self-selected and may not represent users with more severe symptoms. Excluding those with psychiatric conditions may have led to conservative estimates. Reliance on self-report (aside from the Stroop) introduces potential bias; future studies should include clinical interviews and biological verification of PED use. Generalizability is limited by our mostly male, urban Turkish sample. Cultural norms and help-seeking behaviors may influence outcomes. Additionally, the deliberate exclusion of intermediate (1–3 year) users was implemented to ensure clearly distinct short- and long-term exposure profiles; however, this methodological choice may limit the generalizability of findings to individuals with moderate or transitional patterns of PED use. Finally, our non-users had body dissatisfaction but no PED exposure—useful for isolating drug effects, yet likely underestimating group differences. Although we summarized PED use characteristics, all substance and dosage data were self-reported and not biochemically verified. Consequently, the observed psychological differences should be interpreted with caution, as variations in compound potency, stacking practices, and post-cycle therapy could contribute to the heterogeneity of outcomes.

## Conclusion

In conclusion, this study provides a detailed overview of the psychological landscape observed among gym-going PED users. Long-term anabolic steroid use was associated with a broad spectrum of psychological differences—including higher depression and anxiety levels, slower executive performance, and lower social functioning—suggesting potential cumulative effects of extended use. These associations were further shaped by mediating and moderating factors. Emotional distress (depression and anxiety) appeared to mediate the link between PED use and interpersonal functioning, whereas strong self-efficacy and perceived social support were related to more adaptive emotional outcomes. The implication is a nuanced one: while prolonged PED use may correspond with elevated psychological risk, the presence of personal resilience and social resources is associated with better mental health. These findings highlight the importance of multidisciplinary approaches that address both physiological and psychosocial aspects of PED engagement. By attending to users’ psychological well-being and social contexts alongside physical health, professionals can better understand and respond to the complex interplay between performance enhancement and mental health.

## Data Availability

The datasets presented in this study can be found in online repositories. The names of the repository/repositories and accession number(s) can be found below: https://doi.org/10.6084/m9.figshare.29856236.

## Supplementary material

The datasets generated and analyzed during the current study, including supplementary assumption checks, *post hoc* comparisons, mediation/moderation outputs, and regression analyses, are openly available via Figshare at: https://doi.org/10.6084/m9.figshare.29856236.
