# Supplementary figures and images for: Psychological correlates of performance-enhancing drug use: Emotional, cognitive, and social functioning in long-term and short-term users
Source: Front Psychiatry. 2025 Dec 2;16:1710046. doi: 10.3389/fpsyt.2025.1710046 (PMC12705642; doi:10.3389/fpsyt.2025.1710046)

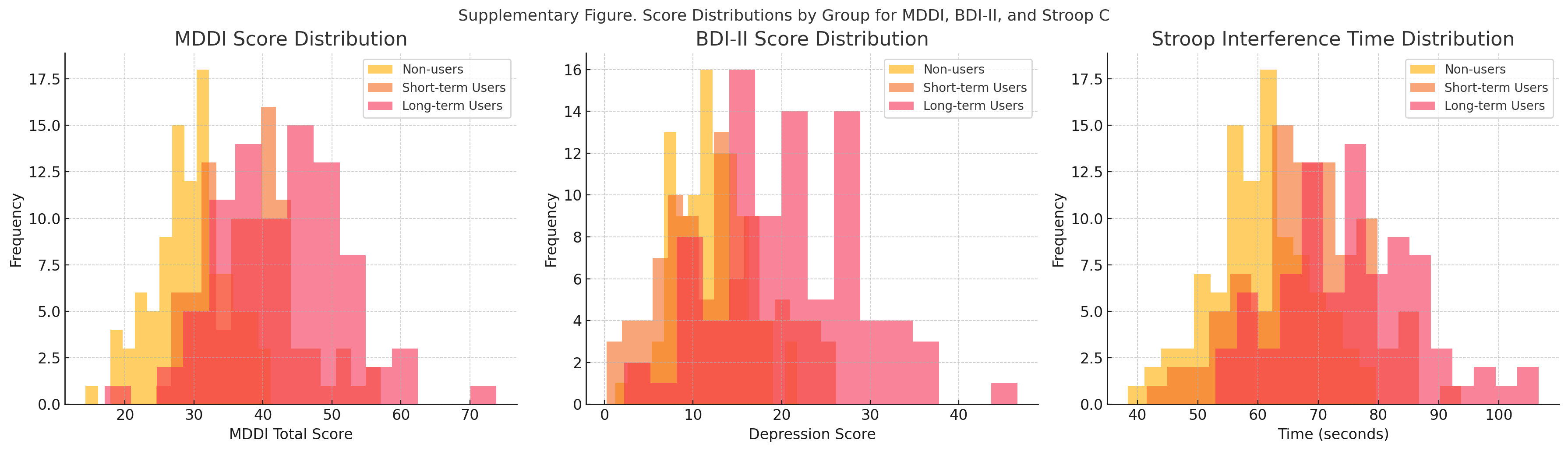

Supplement: Supplementary file 1 [file Image1.png]

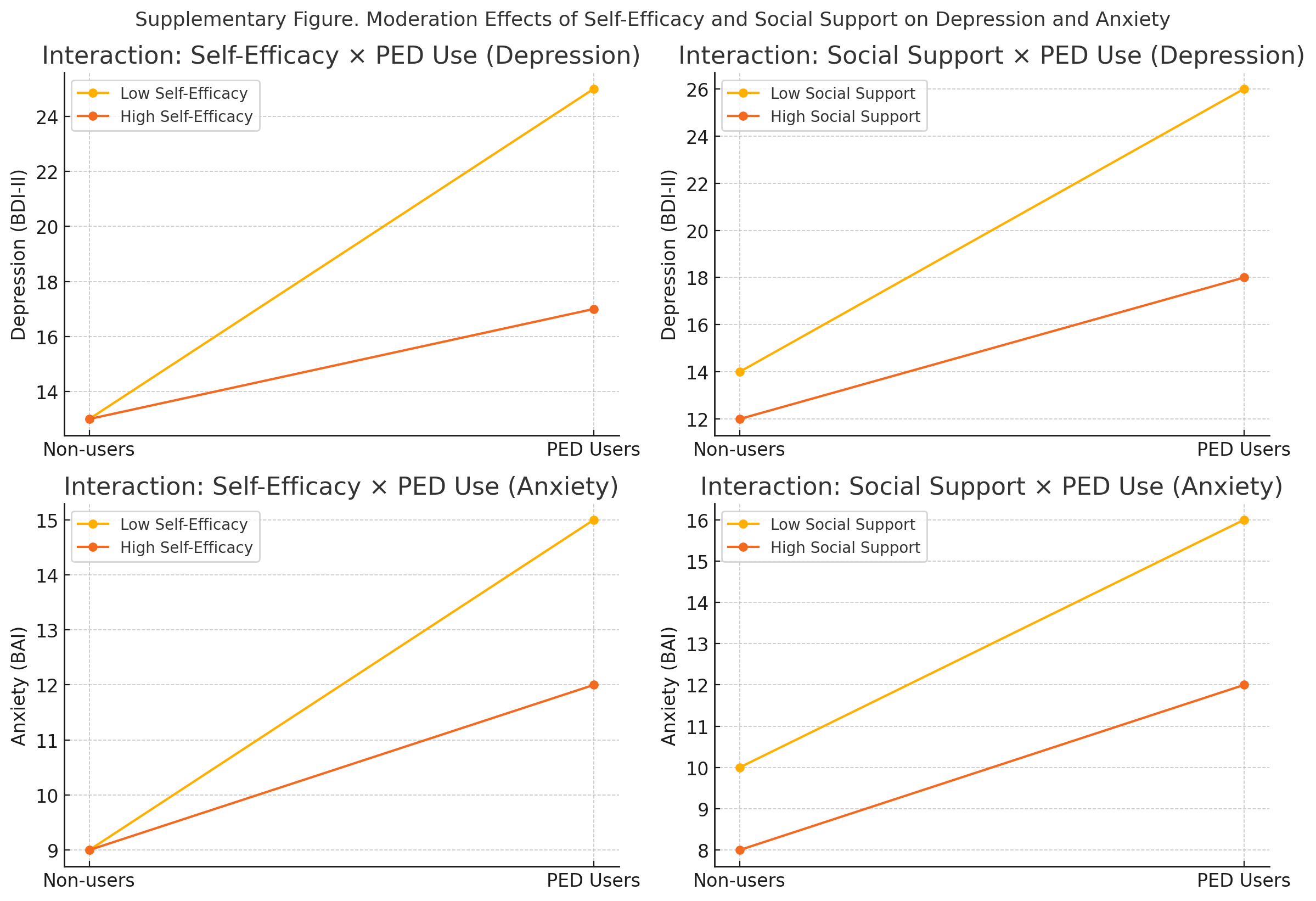

Supplement: Supplementary file 2 [file Image2.png]

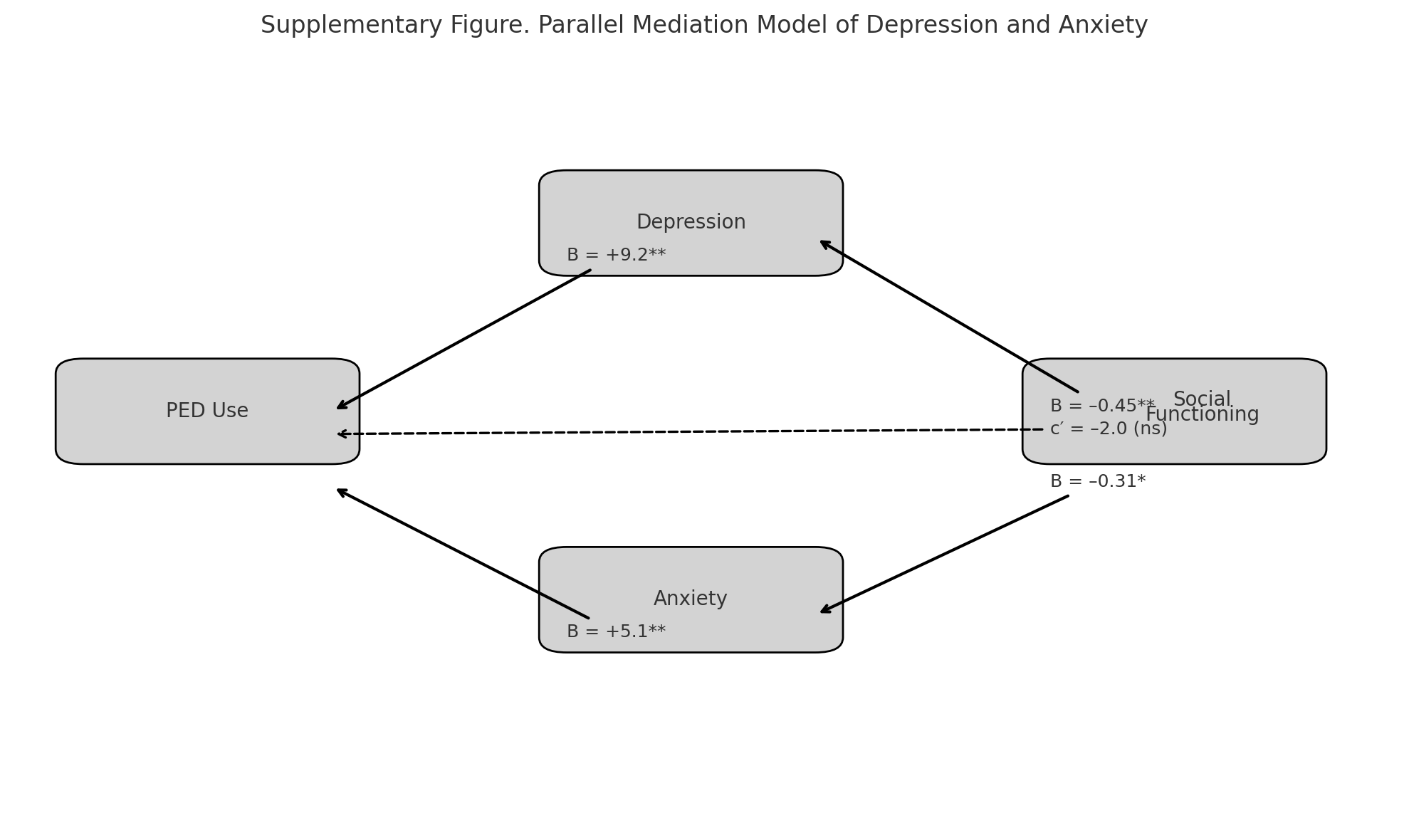

Supplement: Supplementary file 3 [file Image3.png]
