# Supplementary material for: Psychological correlates of performance-enhancing drug use: Emotional, cognitive, and social functioning in long-term and short-term users
Source: Front Psychiatry. 2025 Dec 2;16:1710046. doi: 10.3389/fpsyt.2025.1710046 (PMC12705642; doi:10.3389/fpsyt.2025.1710046)
Supplement: Supplementary file 4 [file Table1.docx]

**Supplementary Table**

**Bootstrapping Results for Mediation and Moderation Models**

| **Model Type** | **Path or Interaction** | **Indirect Effect (B)** | **95% CI (Lower, Upper)** | **Bias-Corrected *p*** | **Effect Size (κ² / ΔR²)** |
| --- | --- | --- | --- | --- | --- |
| Mediation | PED Use → Depression → SASS | –4.21 | [–6.08, –2.60] | < .001 * | 0.15 |
| Mediation | PED Use → Anxiety → SASS | –1.56 | [–2.79, –0.58] | .002 * | 0.08 |
| Moderation | PED Use × GSE → Depression | — | [–0.93, –0.07] | .024 * | 0.02 |
| Moderation | PED Use × MSPSS → Depression | — | [–6.01, –0.41] | .028 * | 0.03 |
| Moderation | PED Use × GSE → Anxiety | — | [–0.84, 0.14] | .090 | 0.01 |
| Moderation | PED Use × MSPSS → Anxiety | — | [–5.10, –0.04] | .037 * | 0.03 |

Bootstrapping results for mediation and moderation models (PROCESS). Mediation models report bias-corrected 95% confidence intervals and kappa-squared effect sizes for indirect effects of depression and anxiety on the relationship between PED use and social functioning (SASS). Moderation models include interaction effects with self-efficacy (GSE) and perceived social support (MSPSS), along with ΔR² values indicating variance explained by moderation. All results are based on 5,000 bootstrap samples.
