# Supplementary material for: Psychological correlates of performance-enhancing drug use: Emotional, cognitive, and social functioning in long-term and short-term users
Source: Front Psychiatry. 2025 Dec 2;16:1710046. doi: 10.3389/fpsyt.2025.1710046 (PMC12705642; doi:10.3389/fpsyt.2025.1710046)
Supplement: Supplementary file 5 [file Table2.docx]

**Supplementary Table**

**Full Regression Outputs for Predicting Depression and Anxiety Among PED Users**

| **Dependent Variable** | **Predictor** | **B (Unstd.)** | **SE** | **β (Std.)** | **95% CI** | **p** | **VIF** |
| --- | --- | --- | --- | --- | --- | --- | --- |
| **BDI-II (Depression)** | Muscle Dysmorphia (MDDI) | 0.50 | 0.08 | 0.42 | [0.34, 0.66] | < .001 * | 1.82 |
|  | Social Support (MSPSS) | –2.00 | 0.60 | –0.25 | [–3.18, –0.82] | .002 * | 1.45 |
|  | Self-Efficacy (GSE) | –0.30 | 0.25 | –0.11 | [–0.78, 0.18] | .120 | 1.35 |
| **BAI (Anxiety)** | Muscle Dysmorphia (MDDI) | 0.35 | 0.08 | 0.33 | [0.19, 0.51] | < .001 * | 1.82 |
|  | Social Support (MSPSS) | –1.50 | 0.50 | –0.22 | [–2.48, –0.52] | .005 * | 1.45 |
|  | Self-Efficacy (GSE) | –0.20 | 0.15 | –0.10 | [–0.49, 0.09] | .180 | 1.35 |

Full regression results predicting depression and anxiety among PED users. The table includes unstandardized (B) and standardized (β) coefficients, standard errors, 95% confidence intervals, and variance inflation factor (VIF) values for multicollinearity assessment. Models controlled for age, gender, and training frequency.
