# Supplementary material for: Psychological correlates of performance-enhancing drug use: Emotional, cognitive, and social functioning in long-term and short-term users
Source: Front Psychiatry. 2025 Dec 2;16:1710046. doi: 10.3389/fpsyt.2025.1710046 (PMC12705642; doi:10.3389/fpsyt.2025.1710046)
Supplement: Supplementary file 6 [file Table3.docx]

**Supplementary Table**

**Assumption Checks for Parametric Analyses**

| **Test Type** | **Variable/Tested Outcome** | **Test Statistic** | **p-value** | **Interpretation** |
| --- | --- | --- | --- | --- |
| Levene’s Test | BDI-II (Depression) | 1.82 | .165 | Homogeneity assumed |
| Levene’s Test | BAI (Anxiety) | 2.01 | .135 | Homogeneity assumed |
| Levene’s Test | SASS (Social Functioning) | 1.55 | .210 | Homogeneity assumed |
| Shapiro-Wilk | BDI-II (Depression) | 0.98 | .240 | Normality not violated |
| Shapiro-Wilk | BAI (Anxiety) | 0.97 | .180 | Normality not violated |
| Shapiro-Wilk | SASS (Social Functioning) | 0.96 | .120 | Normality not violated |
| VIF | Muscle Dysmorphia (MDDI) | 1.82 | — | No multicollinearity |
| VIF | Social Support (MSPSS) | 1.45 | — | No multicollinearity |
| VIF | Self-Efficacy (GSE) | 1.35 | — | No multicollinearity |

Assumption checks for key statistical analyses. Levene’s tests indicated homogeneity of variances for all ANOVAs. Shapiro-Wilk tests did not indicate violations of normality. Variance Inflation Factor (VIF) values for predictors used in regression analyses were all below 2, suggesting no multicollinearity.
