# Supplementary material for: Psychological correlates of performance-enhancing drug use: Emotional, cognitive, and social functioning in long-term and short-term users
Source: Front Psychiatry. 2025 Dec 2;16:1710046. doi: 10.3389/fpsyt.2025.1710046 (PMC12705642; doi:10.3389/fpsyt.2025.1710046)
Supplement: Supplementary file 7 [file Table4.docx]

**Supplementary Table**

**Bonferroni-Corrected Post Hoc Comparisons for Significant ANOVAs**

| **Outcome Variable** | **Group Comparison** | **Mean Difference** | **p (Bonferroni)** |
| --- | --- | --- | --- |
| BDI-II (Depression) | Long-term vs. Short-term | 9 | < .001 * |
|  | Long-term vs. Non-users | 11 | < .001 * |
|  | Short-term vs. Non-users | 2 | .21 |
| BAI (Anxiety) | Long-term vs. Short-term | 3.5 | .002 * |
|  | Long-term vs. Non-users | 4.6 | < .001 * |
|  | Short-term vs. Non-users | 1.1 | .51 |
| MDDI (Muscle Dysmorphia) | Long-term vs. Short-term | 5 | .07 |
|  | Long-term vs. Non-users | 13 | < .001 * |
|  | Short-term vs. Non-users | 8 | < .001 * |
| SASS (Social Functioning) | Long-term vs. Short-term | –5.3 | .045 * |
|  | Long-term vs. Non-users | –10.4 | < .001 * |
|  | Short-term vs. Non-users | –5.1 | < .001 * |
| MSPSS (Social Support) | Long-term vs. Short-term | –0.23 | .12 |
|  | Long-term vs. Non-users | –0.69 | < .001 * |
|  | Short-term vs. Non-users | –0.46 | .002 * |
| Stroop C (Interference Time) | Long-term vs. Short-term | 7 | .015 * |
|  | Long-term vs. Non-users | 15 | < .001 * |
|  | Short-term vs. Non-users | 8 | .08 |
