# Supplementary material for: Psychological correlates of performance-enhancing drug use: Emotional, cognitive, and social functioning in long-term and short-term users
Source: Front Psychiatry. 2025 Dec 2;16:1710046. doi: 10.3389/fpsyt.2025.1710046 (PMC12705642; doi:10.3389/fpsyt.2025.1710046)
Supplement: Supplementary file 8 [file Table5.docx]

**Supplementary Table**

**Zero-Order Pearson Correlation Matrix Among Study Variables**

| **Variable** | **1** | **2** | **3** | **4** | **5** | **6** | **7** |
| --- | --- | --- | --- | --- | --- | --- | --- |
| 1. BDI-II (Depression) | 1.00 | .60 | .50 | –.30 | –.20 | .35 | –.65 |
| 2. BAI (Anxiety) |  | 1.00 | .46 | –.28 | –.18 | .30 | –.60 |
| 3. MDDI (Muscle Dysmorphia) |  |  | 1.00 | –.25 | –.15 | .25 | –.40 |
| 4. MSPSS (Social Support) |  |  |  | 1.00 | .35 | –.20 | .50 |
| 5. GSE (Self-Efficacy) |  |  |  |  | 1.00 | –.10 | .30 |
| 6. Stroop C (Interference Time) |  |  |  |  |  | 1.00 | –.40 |
| 7. SASS (Social Functioning) |  |  |  |  |  |  | 1.00 |

**Note.** All values are Pearson’s r. Higher scores on BDI-II, BAI, MDDI, and Stroop C reflect greater symptom severity or impairment; higher scores on MSPSS, GSE, and SASS reflect greater functioning or protective factors. Correlations above .30 or below –.30 are considered moderate to strong.
