# Supplementary material for: Psychological correlates of performance-enhancing drug use: Emotional, cognitive, and social functioning in long-term and short-term users
Source: Front Psychiatry. 2025 Dec 2;16:1710046. doi: 10.3389/fpsyt.2025.1710046 (PMC12705642; doi:10.3389/fpsyt.2025.1710046)

CONSORT-style flow diagram depicting participant recruitment, screening, exclusions, and final sample composition.
Of the 436 gym-goers initially approached, 125 were classified as long-term users (>3 years), 142 as short-term users (<1 year), and 169 as non-users. Following assessment attendance and exclusion screening (e.g., psychiatric medication, substance use, inconsistent responses), 285 participants were retained for analyses: 87 long-term users, 95 short-term users, and 103 non-users.


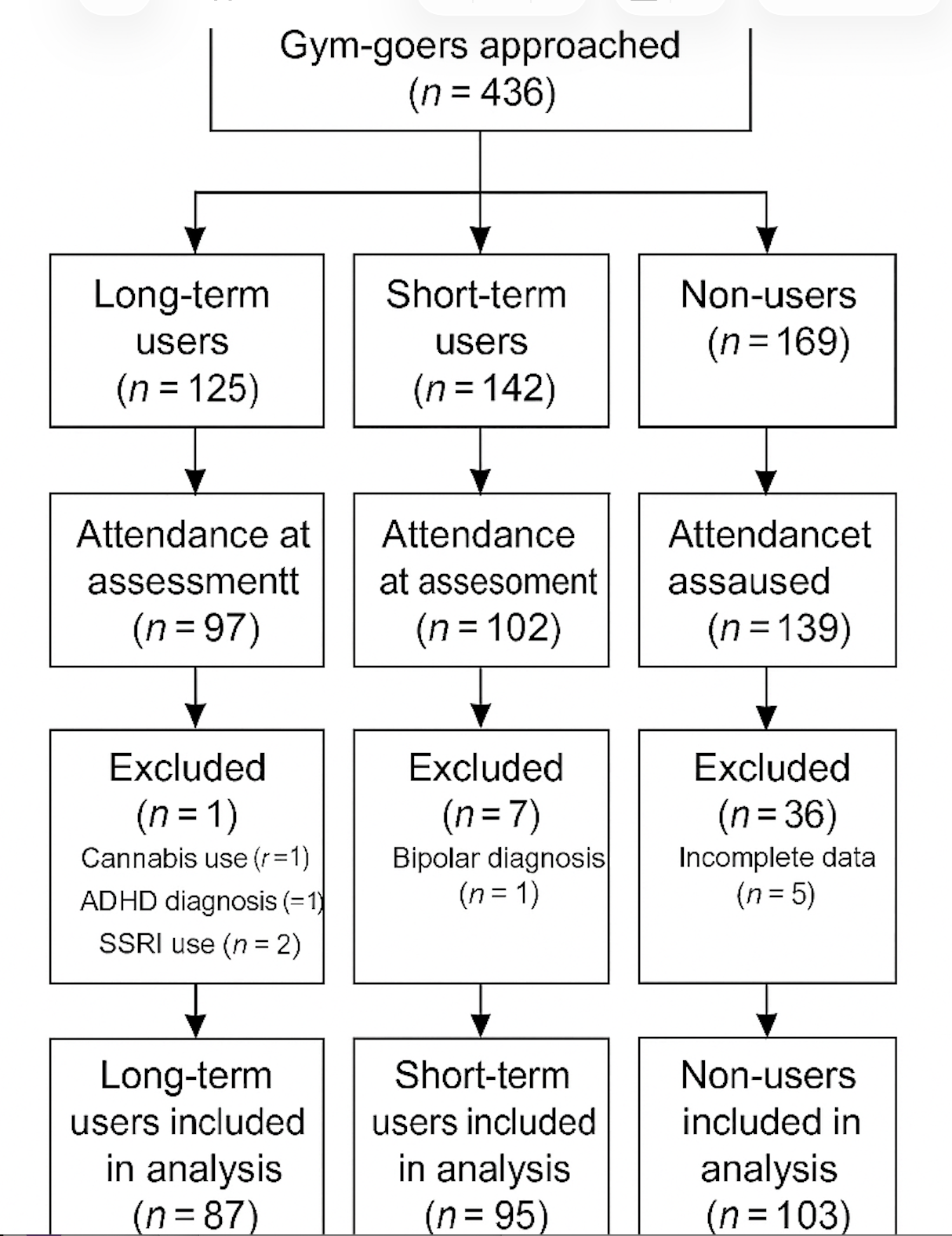

Supplement: Supplementary file 9 [file Table6.docx]
